# The impact of neuroscience education therapy, behavioral economics, and digital navigators on patient migraine treatment adherence to a mobile health application: a prospective pilot randomized controlled trial

**DOI:** 10.1038/s44277-024-00021-w

**Published:** 2025-01-08

**Authors:** Mia T. Minen, Erin K. Waire, John Torous, Jessica Fishman, Richard B. Lipton, Scott W. Powers

**Affiliations:** 1 Departments of Neurology and Population Health, NYU Langone Health, New York, NY USA; 2 Department of Neurology, NYU Langone Health, New York, NY USA; 3 Department of Psychiatry, Beth Israel Deaconess Medical Center, Harvard Medical School, Boston, MA USA; 4 Message Effects Lab, University of Pennsylvania, Philadelphia, PA USA; 5 Department of Neurology, Albert Einstein College of Medicine, New York, NY USA; 6 Department of Pediatrics, University of Cincinnati College of Medicine, Cincinnati, OH; Division of Behavioral Medicine and Clinical Psychology, Cincinnati Children’s Hospital, Cincinnati, OH USA

**Keywords:** Migraine, Randomized controlled trials

## Abstract

Mobile health (mHealth) tools can be used to deliver nonpharmacologic therapies to patients with migraine. However, mHealth studies often report poor treatment adherence. Neuroscience Education Therapy (NET), behavioral economics, and Digital Navigators have the potential to increase treatment adherence and thereby improve remote migraine self-management. We conducted a 6-month prospective pilot randomized controlled trial testing if a multi-component package of behavioral interventions increased treatment adherence among patients using one of two different mHealth migraine self-management programs (low-intensity program consisting only of a headache diary versus high-intensity program consisting of a headache diary and behavioral exercises). Our outcomes were the number of diary entries and behavioral exercises completed/week captured via back-end analytics of the mHealth application. We also compared our adherence data at 90-days (a secondary endpoint to assess the durability of the effect) with adherence data from similar published studies without the adherence-enhancing package. We enrolled 26 participants (n = 15 low intensity group, n = 11 high-intensity group). During the 6-week intervention period, we had a median of 7 headache diary entries/week in both groups and a median of 6 days/week of behavioral exercises in the high-intensity group. The rate of adherence with the adherence-enhancing package included was 2.9-8x higher compared to the median rates of the behavioral exercises to historical controls. With use of NET, behavioral economics, and digital navigators, participants achieved higher levels of adherence to both self-management programs compared to prior remote migraine self-management studies. Therefore, these tools may be beneficial to improving adherence to migraine self-management programs.

## Lay Summary

Mobile health studies often face adherence and engagement issues. In this study, Neuroscience Education Therapy (NET), behavioral economics (BE), and Digital Navigators were used with the intention to increase adherence to a remote migraine self-management program.

## Introduction

Migraine is the second most disabling condition globally in terms of disability-adjusted life years [1, 2]. In the United States, 15.9% of the adult population is affected by migraine and severe headaches [3] With the high prevalence and disability of migraine, access to care is crucial, yet there is a shortage of trained providers leaving many patients with migraine undertreated [4] This under treatment of migraine occurs even though there are effective pharmacologic and nonpharmacologic treatments [5–7] Compounding to the under treatment is poor adherence to therapies, due to costs, insurance barriers, difficulty finding specialty providers, and the time and effort required to attend in-person nonpharmacologic appointments [8–10] Novel treatment options to overcome these access barriers are needed for treating migraine [11–14].

Mobile health (mHealth) interventions for migraine have the potential to allow patients to access the interventions wherever and whenever it is most convenient for them, thus overcoming some of the known challenges of evidence-based in-person delivered behavioral therapies. However, mHealth interventions have their own adherence issues; a systematic search of 93 mental health applications (apps) with at least 100,000 installs, revealed a median open rate of 4.0% (IQR 4.7%), meaning that only 4.0% of people who installed the app used it daily [15] Further, retention rates are low; a sharp decline (>80%) was observed in app open rates in the first 10 days, and the median app retention rate at 15 days was 3.9% (IQR 10.3%) and at 30 days was 3.3% (IQR 6.2%). Similar findings are reported in the headache literature; in a commercial app study, about one-third (32.4%) of participants completed their headache diary for 90 days, and half of the participants completed ≤ 34 of the 90 days [16] However, higher use of mHealth interventions has been shown to improve clinically important migraine outcomes [17–19] Such interventions, like those of RELAXaHEAD and the HeartMath Inner Balance Sensor, which use Grade A evidence-based treatments for migraine, such as relaxation and biofeedback, have been shown to benefit patients with clinically important outcomes *if* patients adhere to treatments [18]. However, as with most mHealth studies, studies have shown poor adherence for the majority of users [18–21]. Thus, improving adherence (i.e., user engagement) is essential among mHealth studies for migraine.

Enhancements to improve engagement in mHealth studies may include the use of behavioral economics (BE) and the use of digital navigators, and in migraine research, the use of neuroscience education therapy (NET) has been found to help with adherence to migraine treatment more generally. Behavioral economics, defined as the intersection between behavior change (e.g., decision-making) and economic principles [20], has shown promise in improving adherence in mHealth studies [21, 22] These principles can be used to facilitate behavior and lifestyle changes with the intent to improve adherence to health-promoting activities. Although financial compensation is often provided to study participants, poor adherence remains a common occurrence. Loss aversion, the principle that monetary risk is more motivating than monetary gain, has been effective at improving medication adherence in mHealth studies [23, 24] Self-monitoring is a major component of mHealth. The principle of regret aversion has shown to be beneficial at improving adherence to self-monitoring in mHealth studies [25] Additional principles of behavioral economics, such as personalization, pre-commitment, “fresh” start, and accountable justification, can be implemented with the intent to increase study adherence.

With the expanding use of mHealth, Digital Navigators are becoming increasingly important with the primary role to provide digital support to patients [26]. Standardizing the qualifications to obtain this role is underway (i.e., training curriculum) [26, 27]. The Digital Navigator model is designed to address challenges related to mHealth patient engagement with published training modules [28], numerous implementation examples [27, 29, 30], and a 2023 review paper on the uses of the role [26]. Digital Navigators can play an essential role in simplifying the evaluation, selection, and use of health-related smartphone apps for clinicians and patients [31]. The Digital Navigator can also assist with data interpretation for the clinician and/or the patient after an app is used [32]. In mHealth research, Digital Navigators can provide ongoing technical support and guidance to participants to increase their understanding of the app features. Adherence and app usage may improve as participants may feel more confident and comfortable with the app as a result of the experience with the Digital Navigator [26, 33]. In a research context, Digital Navigators offer a standardized approach to training and human support, that can help ensure research results are replicable and reproducible. Digital Navigators support mHealth research seeking to understand the effect size of apps, human support, and specific behavioral techniques (in this case behavioral economics). While core aspects of the Digital Navigator’s role are standardized, there is also flexibility for different delivery modes (e.g., calls, email, face-to-face, text messages) that provide necessary customizations [26].

Neuroscience education therapy (NET) is an evidence-based treatment for pain conditions [34], and has the potential to improve adherence to behavioral interventions because it breaks down why these interventions may work for individuals in a more comprehensible way. Individuals learn the neuroscience behind why the treatments are effective, and thus may better understand why it is important to adhere to the recommended therapies. One migraine-related study found that those who received NET were 96% (48/60) compliant with their migraine preventive medications compared to those who did not receive the education (58.5%, 24/41) [35]. Findings from migraine-related studies implementing NET in addition to common pharmacologic treatments have shown reductions in migraine disability (MIDAS), migraine days, duration, intensity, and acute medication use [34]. In combination with physiotherapy, NET has also been shown to reduce migraine frequency [36].

This study aims to determine the collective impact of behavioral economics, Digital Navigators, and NET on patient adherence to remote migraine self-management programs using a mHealth application. We hypothesize that patient adherence to the remote migraine self-management programs using a mHealth application will be higher compared to historical controls.

## Methods

The NYU Langone Health Institutional Review Board approved this study. This is a pilot study for the following study registered on ClinicalTrials.gov (NCT06077838). Study participants completed electronic informed consent via REDCap.

A prospective pilot randomized controlled trial (RCT) compared adherence of two interventions: a low intensity (~1–2 minutes/day) program consisting of a daily headache diary and a high intensity (~15 minutes/day) program consisting of a daily headache diary and behavioral exercises. Participants were randomized to one of the two migraine self-management programs. All participants were blinded to the behavioral intervention prior to randomization. Those randomized to the high-intensity group received information about the behavioral exercises during enrollment. Participants in both programs received the multi-component adherence-enhancing intervention (i.e., NET, behavioral economics, and Digital Navigators). We compared adherence in the low-intensity program versus high-intensity program as well as to previously published studies without the multi-component adherence-enhancing intervention. We sought to determine if behavioral economics, specifically financial incentives, “fresh” start, loss aversion, pre-commitment, accountable justification, and regret aversion, as well as the use of NET and Digital Navigators, improve patient adherence to mHealth-based migraine self-management programs compared to prior mHealth migraine self-management studies.

### Participant Recruitment and Inclusion/Exclusion Criteria

Study recruitment and enrollment were completed remotely in November and December 2023. Potentially eligible participants were recruited from NYU Langone Health primary care practices.

An Epic MyChart message describing the study was sent to potentially eligible patients in primary care practices across NYU Langone Health. The message contained study information and a preliminary screener survey. Potentially eligible and interested patients were able to complete the preliminary screener and/or report interest in the study. The study team screened the interested patients to determine eligibility. In addition to the initial MyChart recruitment message, potentially eligible and interested patients received up to five phone calls and two emails informing them of the study and inviting them to participate.

Inclusion criteria included English speaking, ages ≥16 years of age, meeting International Classification of Headache Disorder (ICHD)-3 migraine criteria, 4-29 headache days a month, and a score of ≤75 on the Role Function-Restrictive domain of Migraine Specific Quality of Life Questionnaire version 2.1. Potential participants were excluded if they used Cognitive Behavioral Therapy, Biofeedback, or other Relaxation Therapy for migraine in the past year, had a history of alcohol or other substance abuse, current opioid and/or barbiturate use, a score >15 on the Patient Health Questionnaire-8, indicating moderately severe to severe depression, unable or unwilling to follow a treatment program that relies on written and audio-taped materials, not having a smartphone, and being pregnant.

### Enrollment

Participants were enrolled in the study for 6 months. At enrollment, participants were randomized to either a low-intensity or high-intensity migraine self-management program via REDCap. Randomization was stratified by headache frequency (episodic vs. chronic migraine) and sex at birth (3:1 ratio of females to males). Participants were blinded to the behavioral component of this study prior to enrollment. Those randomly assigned to the high-intensity program were provided with additional details about the behavioral exercises, more specifically, participants in the higher-intensity program were asked to complete a daily headache diary using a mHealth application (RELAXaHEAD) and to listen to daily audio recordings of migraine behavioral self-management exercises in the app (RELAXaHEAD version with behavioral exercises). Participants in the low-intensity program completed a daily headache diary using a mHealth application (RELAXaHEAD version without behavioral exercises). Adherence to the migraine self-management programs was measured by the number of diary entries completed per week and the number of days of behavioral exercises completed per week. Participants were asked to complete the headache diary and behavioral exercises (if in the high intensity program) daily for 6 weeks. After 6 weeks, participants were no longer asked or compensated to use the app daily and could choose whether or not they wanted to continue using the app for migraine self-management.

### Adherence Enhancements

As shown in Table 1, a multi-component approach was used to enhance adherence of both programs, including NET, principles of behavioral economics, and the use of Digital Navigators.

**Table 1:** Adherence Enhancements for the Low-Intensity and High-Intensity Programs.

Mechanisms to Enhance Adherence	Rationale	Adaptation into Study Design
**Behavioral Economics (BE)**	•BE approaches can improve engagement with mHealth programs and experts call for adapting BE in more app study designs [45]. •Approaches such as pre-commitment [46], accountability [47], justification [39], and financial incentives [48] can improve participant adherence [39, 49–52]	•Remote Monitoring allows for the principles of BE to be integrated into the study design as described in detail in Table 2.
**Neuroscience Education Therapy (NET)**	•NET is a treatment which involves education on the neurophysiology of pain to improve patients understanding of migraine, impacting pain perception, migraine disability, catastrophization, avoidance, and fear [34, 53]. •Patients can understand neurophysiology of pain if presented non-esoterically [54]. •NET topics include: “pain does not equate to injury, pain is generated in the brain, perception, genetics, reward systems, fear, brain plasticity, and placebo and nocebo effects.” [55]. •NET can be combined with evidence-based behavioral therapies for migraine [55]. •NET has been shown to promote adherence to migraine preventive medications [35]. Participants in the NET group were 1.6 times more compliant with migraine preventive medications; 96% (48/50) were compliant with preventive medications compared to 58.5% (24/41) in the control group [35]. •Participants in focus groups about migraine mind-body intervention (MBI) research studies indicated that they want to understand why the MBIs work for migraine [56]	•The pre-recorded video at the enrollment session shown to both groups included expert headache specialists explaining: (1) why migraine occurs-including the pathophysiology of migraine, migraine triggers; (2) the importance of self-management; and (3) the reasons why MBIs work (high intensity program only). •Participants were informed that prior studies revealed that those who practiced the behavioral exercises ≥ 2 days/week (i.e., high users) gained benefit in their migraine frequency compared to those who did not.
**Digital Navigators (DN)**	•DNs have been trained to be low-entry positioned staff who can help patients with the technology, engagement, etc. of digital tools [28, 57–60]. •A curriculum consisting of 10 hours of training has been designed for DN training [28]. •DNs are considered a low cost, scalable option for potential use globally [60]	•Study team members involved in enrollment and follow-up were trained by Dr. John Torous, the developer of Digital Navigators.

As shown in Table 2, principles of behavioral economics were implemented throughout the study for both programs. Personalization and pre-commitment were used to improve participant engagement with the headache diary and/or behavioral exercises through the provision of personalized goals and the adaptation of goals based on performance [37, 38]. During enrollment, participants were asked their level of intention to use the app most days per week (6 of 7 days) during the 6-week intervention period. At the end of each week during the intervention period, participants were asked how many days they planned to commit to the program for the following week via email.

**Table 2:** Principles of Behavioral Economics.

Nudge	Rationale	Adaptation into Study Design
Financial Incentives/Fresh Start/Loss Aversion	•People respond more to losses than to gains. Getting people to pre-commit and put money at risk is an effective tool in select contexts [37, 39, 40]. •Since we know people using behavioral interventions can “fall off the wagon” [40], financial incentives were replenished weekly to leverage the tendency that increased motivation to complete a goal when it is associated with a “landmark” time point.	•Participants were given $14 in a “bank account” at the start of each week of the 6-week intervention period. •Participants were told that for every day they do not participate, they will lose $2.
Personalization/ Pre-commitment	•Programs are more effective when individual baselines and personalized goal setting (i.e., goals may increase in demand or may be adapted based on current performance) are incorporated. For example, implementing personalized step plans with wearable devices for patients with ischemic heart disease can significantly change behaviors when only providing the device is unlikely to result in any significant change [37, 38]	•At the enrollment session, both groups were asked to commit to daily diary usage. •Each week RELAX users were asked to commit to a personalized number of days to complete the diary and behavioral exercises per week and then sent a mid-week text message with their progress towards that goal.
Accountable justification/ Regret aversion	•Justification motivates participants to complete future sessions if they feel regret not meeting present goals [22]	•Participants were asked to provide justification for every 3 days of missing diary data.

Financial incentives, “fresh” start, and loss aversion were also implemented. Individuals tend to respond more often to monetary risk than monetary gain. Therefore, the monetary risk may motivate daily participation to avoid any financial loss [37, 39, 40]. At the beginning of each week of the 6-week intervention period, participants were allocated $14. For each day they did not participate in the program, they lost $2. At the end of each week, participants received an email with the total number of days they participated that week, the amount of money earned, and a reminder that the next week is a fresh start.

Research assistants were provided with two days of in-person Digital Nnavigator training covering the core components of digital health trouble shooting, digital health tool evaluation, and digital health engagement support. In this study, text messaging was selected as the mode of communication between the digital navigator and participants. Participants received text messages for every 3 days of missed participation of the program. The text messages from the Digital Navigator included a notification of missed participation, encouragement to continue participation, and requested the participant to provide reasoning for missing days. Participants in the high-intensity program received an additional text message with efficacy data for the behavioral exercises. The text messages use accountable justification and regret aversion with the expectation that it motivates participants to complete future days as they may experience regret for not achieving daily participation [22].

### Data analysis

Descriptive statistics were reported for baseline data, including demographics and adherence to a daily headache diary and behavioral exercises. No statistical tests were conducted as this was an under-powered study with a small sample size. We collected 90-Day adherence levels in this study and compared them to prior mHealth migraine studies.

## Results

As shown in Table 3, a total of 26 participants were enrolled in the pilot study (low intensity program, n = 15; high intensity program, n = 11). The mean age of participants was 38.6 years (range 22-59, SD 10.2). All participants identified as female (n = 26). The majority of participants identified as White or Caucasian (n = 18, 69.2%) followed by other racial groups or prefer not to answer (n = 5, 19.2%) and African American (n = 3, 11.5%). Almost one quarter (23.1%) of participants identified as Hispanic or Latino (n = 6).

**Table 3:** Baseline Demographics (N = 26)

	Low Intensity Program (n = 15)	High Intensity Program (n = 11)	Total (N = 26)
Age			
Mean (SD)	41.1 (10.8)	35.1 (8.6)	38.6 (10.2)
Median (IQR)	39 (13.0)	38 (20.0)	38.5 (11.75)
Gender n (%)			
Female	15 (100)	11 (100)	26 (100)
Race n (%)			
White/Caucasian	11 (73.3)	7 (63.6)	18 (69.2)
African American	2 (13.3)	1 (9.1)	3 (11.5)
Other/prefer not to answer	2 (13.3)	3 (27.3)	3 (11.5)
Ethnicity n (%)			
Not Hispanic or Latino	13 (86.7)	7 (63.6)	20 (76.9)
Hispanic or Latino	2 (13.3)	4 (36.4)	6 (23.1)

### 6-week Intervention Period

Among all pilot participants, the median headache diary entries per week was 7.0 (IQR 1.0; mean, 6.3 ± 1.6). The mean headache diary entries per week differed slightly between those in the low-intensity and high-intensity programs. The mean headache diary entries per week was 6.6 ± 0.9 in the low-intensity program and 5.8 ± 2.2 in the high-intensity program.

**Fig. 1:**
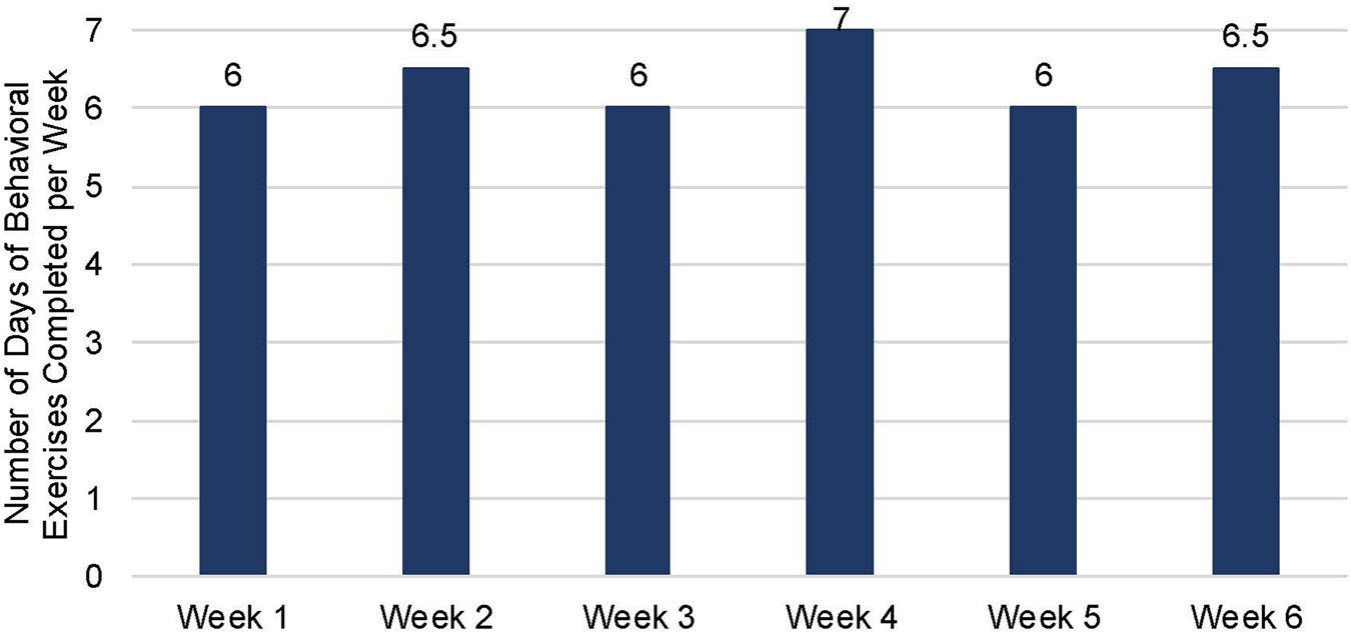
Median Rate of Behavioral Self-Management Exercises per week during 6-Week Intervention Period (n = 11).

Among those in the high-intensity program (n = 11), participants completed a median of 6.0 (IQR 4.0) days of behavioral exercises per week (mean, 5.1 ± 2.3), with the weekly median adherence ranging between 6.0-7.0 days a week. See Fig. 1 for the median rate of days of behavioral exercises completed per week. Nearly three quarters (72.7%, 8/11) of participants completed an average of ≥4 days per week during the 6-week intervention period (i.e., high users).

A median of 34.0 (IQR 14.5) days of behavioral exercise (mean, 28.3 ± 13.4) and 41.0 (IQR 4.8) diary entries (mean, 36.4 ± 10.8) were completed during the intervention period. Participants in the low-intensity program had a median of 41.0 (IQR 3.0) diary entries (mean, 39.5 ± 4.4) while participants in the high-intensity program completed a median of 41.0 (IQR 14.5) diary entries (mean, 32.1 ± 15.1).

### 90-Day Study Period

During the 90-day study period, participants completed a median of 5.0 (IQR 5.3) days of behavioral exercises per week (mean, 3.8 ± 2.8). Among participants in both programs, a median of 7.0 (IQR 5.0) diary entries per week (mean, 4.8 ± 2.9) were completed (low-intensity program, median, 7.0 (IQR 4.9), mean, 4.9 ± 2.9; high-intensity program, median, 6.0 (IQR 5.3), mean, 4.7 ± 2.9).

During the 90-day study period, a median of 40.0 (IQR 41.5) days of behavioral exercises (mean, 42.3 ± 26.6) and a median of 58.5 (IQR 43.0) diary entries (mean, 59.0 ± 27.2) were completed. Participants in the low-intensity program had a median of 59.0 (IQR 40.0) diary entries (mean, 63.1 ± 22.0) while participants in the high-intensity program completed a median of 57.0 (IQR 56.0) diary entries (mean, 53.4 ± 33.5).

**Fig. 2:**
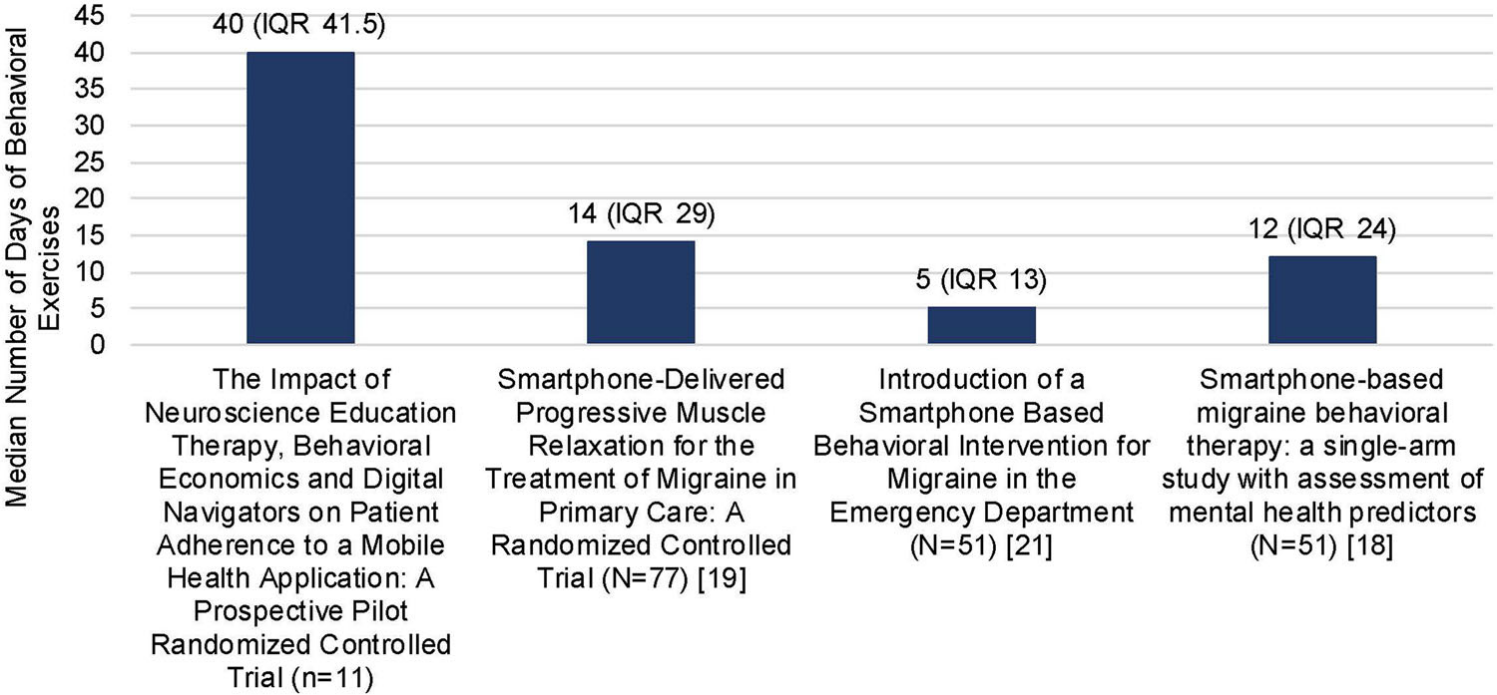
Comparison of Median Rates of Daily Behavioral Exercises to Prior RELAXaHEAD Studies during 90-Day Period.

As shown in Fig. 2, a comparison of the median rates of daily behavioral exercises to historical controls resulted in 2.9-8x higher rates of adherence with the addition of the adherence-enhancing package.

## Discussion

In this prospective pilot RCT, we found that participants achieved high levels of adherence to both the low- and high-intensity self-management programs compared to prior migraine mHealth self-management programs (by our team and others). These results suggest that the use of behavioral economics, NET, and Digital Navigators may be effective at increasing treatment adherence for migraine.

Prior studies have assessed the feasibility and acceptability of the same migraine self-management program (RELAXaHEAD with and without behavioral exercises). A prior RCT of RELAXaHEAD in primary care implemented financial incentives for days of use (but did not use loss aversion) and used reminder notifications for every 3 days of non-participation (but did not use accountable justification or regret aversion); participants completed 2-4 days of migraine self-management per week [19]. Similarly, in a prior RCT of RELAXaHEAD in the emergency department (included financial incentives for days of use; no implementation of behavioral economics), among those who completed ≥1 day per week of diary use, the migraine self-management program was completed 2.3 ± 2.4 days per week [41]. Adherence was also relatively low in a single-arm study of RELAXaHEAD within the neurology outpatient setting with only 25% of participants completing the migraine self-management program ≥ 2 days per week [18]. In this pilot study, implementing NET, behavioral economics, and formally trained Digital Navigators, may have motivated participants to improve app engagement resulting in higher adherence in this study compared to the prior studies.

Of note, adherence to daily diary entries in the low-intensity program was higher compared to the high-intensity program. The additional daily behavioral exercises in the high-intensity program may have resulted in intervention fatigue and thereby lower adherence to the daily diary entries. Intervention fatigue can impact our ability to determine the dose-response relationship and overall effectiveness of the intervention, yet adherence rates were still dramatically higher than prior historic studies. A better understanding of individual factors, with the potential to impact adherence, may provide valuable insight into who would benefit most from the intervention.

Adherence is a common concern among clinicians and researchers with the ability to impact patient health and data integrity. In this study, adherence to the migraine self-management program was higher compared to adherence in other mHealth migraine studies which demonstrates the potential benefits of the implementation of NET, behavioral economics, and Digital Navigators in mHealth research. In a study evaluating heartrate variability biofeedback (HeartMath) for migraine, those in the high user group had improvements in migraine-specific quality of life (12-point change in MSQv2 at day 30 (*p* = 0.010)) while those in the low user group had no significant change (*p* = 0.765)) [17]. A naturalistic study of a commercially designed headache app, the N1-Headache app (formerly Curelator), found that just 32.4% (505/1561) of participants achieved 90-day adherence [42].

Behavioral economics have previously been used in other areas of mHealth but not headache medicine. It has been shown to be effective in mHealth studies involving behavior change [43], self-monitoring [25], and medication adherence [24] among different populations. Our results show that behavioral economics may also be effective in mHealth studies among patients with migraine. Further research is necessary to determine the extent to which behavioral economics can influence adherence and user engagement in mHealth studies for headache.

The implementation of Digital Navigators is a novel approach to improve adherence in mHealth headache studies. The Digital Navigator is intended to improve participants’ understanding of the app features in order to increase confidence and comfort in their ability to use the app [26]. Additional research is needed to determine the effect of Digital Navigators on adherence and user engagement in mHealth research. Given the standardized nature of the training, this is highly feasible and scalable [44].

### Strengths

This study was the first to assess a comprehensive behavioral enhancing package to improve adherence. The study was successfully conducted in a fully remote manner and patients were blinded to the behavioral intervention in the study. Many migraine studies are not diverse; about one third of participants did not identify as White/Caucasian.

### Limitations

As this is a pilot study, the sample size is relatively small (N = 26). As such, the study was not powered to detect differences and only descriptive statistics could be reported (no statistical tests). Also, given we wanted to optimize adherence, we used an adherence enhancing multiple component strategy. We do not know whether individual components were most effective in improving adherence. As we were assessing the feasibility of the adherence strategy, we do not yet know whether these strategies improve clinical outcomes related to migraine. In terms of generalizability, participants had to indicate that they were at least neutral about participating in a migraine mHealth based study. This would likely be a similar demographic to those who would download a migraine app on their own. However, future work will need to include a larger sample size so that more conclusions can be drawn regarding generalizability.

## Conclusion

Multiple adherence enhancing tools like behavioral economics, Digital Navigators and NET may be beneficial to improving participant adherence to mHealth-based migraine self-management programs. Future work will thus consist of a large-scale Phase 3 trial examining whether smartphone-based behavioral exercises using RELAXaHEAD improves migraine related quality of life and disability and will use these adherence enhancing components to boost adherence, which in turn we expect to improve the clinically important migraine outcomes. If there is improvement in the clinical outcomes, additional work can then assess the effect of the individual components of this multi-component adherence enhancing strategy. In addition, other mHealth studies for migraine can incorporate such adherence enhancing strategies to boost adherence and hopefully improve clinically important outcomes.

## Supplementary Information

CONSORT Flow chart

## Data Availability

Anonymized data not included in this article will be made available by request from any qualified investigator and upon execution of a data sharing agreement.
