# Supplementary figures and images for: The impact of neuroscience education therapy, behavioral economics, and digital navigators on patient migraine treatment adherence to a mobile health application: a prospective pilot randomized controlled trial
Source: NPP Digit Psychiatry Neurosci. 2025 Jan 8;3:2. doi: 10.1038/s44277-024-00021-w (PMC12510445; doi:10.1038/s44277-024-00021-w)

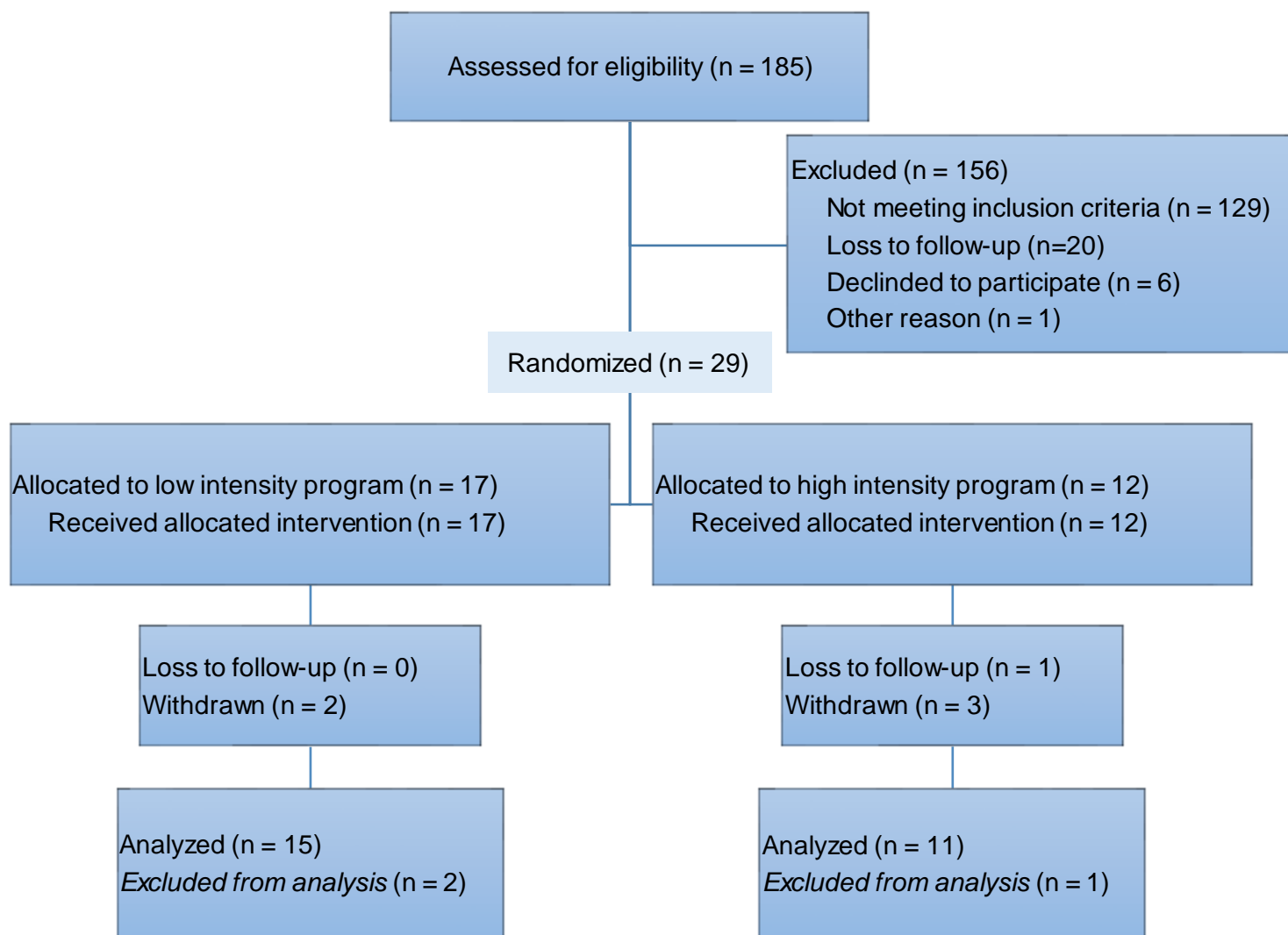

Supplement: Supplementary file 1 — CONSORT Flow chart [file 44277_2024_21_MOESM1_ESM.pdf]
